# Mechanisms of Different Motor Neurons in the Occurrence of Spasticity After Spinal Cord Injury: A Narrative Review

**DOI:** 10.3390/ijms26115162

**Published:** 2025-05-28

**Authors:** Han Gong, Ze-Yan Zhang, Zhi-Xuan Duan, Xin-Ao Mao, Yuan-Yuan Wu, Jia-Sheng Rao, Xiao-Xia Du

**Affiliations:** 1 Beijing Key Laboratory for Biomaterials and Neural Regeneration, National Medical Innovation Platform for Industry-Education Integration in Advanced Medical Devices (Interdiscipline of Medicine and Engineering), School of Biological Science and Medical Engineering, Beihang University, Beijing 100191, China; gonghan2024@buaa.edu.cn (H.G.); yuanywu@buaa.edu.cn (Y.-Y.W.); 2 School of Rehabilitation Sciences and Engineering, University of Health and Rehabilitation Sciences, Qingdao 266113, China; 3 Beijing International Cooperation Bases for Science and Technology on Biomaterials and Neural Regeneration, Beijing Advanced Innovation Center for Biomedical Engineering, Beihang University, Beijing 100191, China; 4 School of Rehabilitation, Capital Medical University, Beijing 100086, China; hyzhangzeyan@163.com (Z.-Y.Z.); dmooou@gmail.com (Z.-X.D.); maoxinao0713@163.com (X.-A.M.); 5 China Rehabilitation Research Center, Beijing 100086, China

**Keywords:** spinal cord injury, spasticity, motor neuron, persistent inward currents

## Abstract

Spasticity is a common complication after spinal cord injury (SCI) that significantly diminishes quality of life and complicates daily management. As a hallmark of upper motor neuron lesions, spasticity emerges through a complex post-injury process involving the resolution of spinal shock, an imbalance between excitatory and inhibitory signaling, and maladaptive neuronal plasticity, leading to hyperreflexia and chronic spasticity. Severe spasticity frequently results in pain, sleep disturbances, and marked functional impairments. This review systematically integrates motor neuron alterations with corresponding muscle manifestations, providing a comprehensive analysis of the brain–spinal cord–muscle pathway in spasticity pathogenesis. Through an in-depth analysis of the pathological and physiological changes in motor neurons post-SCI, this review offers a novel perspective that unveils the intrinsic mechanisms underlying spasticity formation, thereby establishing a robust theoretical foundation for developing targeted therapeutic strategies.

## 1. Introduction

Muscle spasticity [1,2], a prevalent complication affecting over 70% of patients with spinal cord injury (SCI), significantly diminishes their quality of life and presents considerable challenges in daily management [3]. As a hallmark clinical manifestation of upper motor neuron lesions [4], spasticity develops through a complex pathological process following SCI.

Initially, patients enter a state of spinal shock, characterized by diminished muscle tone, suppressed reflexes, and the absence of tendon reflexes below the level of injury, accompanied by muscle paralysis and flaccid paralysis [5]. However, in the subsequent days to months, this state gradually resolves, marked by the restoration of reflexes and an increase in muscle tone. This transition is largely attributed to the abnormal neuronal plasticity that occurs post-SCI, where the balance between excitatory and inhibitory signals is disrupted [6]. Consequently, spinal reflexes are released from inhibitory control, resulting in excessive excitation. As the pathological process advances, persistent inward currents (PICs) in spinal motor neurons are progressively amplified, leading to motor neuron excitability that surpasses normal thresholds. This phenomenon culminates in hyperreflexia, sustained flexor muscle contractions following brief stimuli, and the clinical manifestation of muscle spasticity [7,8].

The significance of spasticity following SCI is characterized by a dual nature [9]. Mild spasticity is generally considered to have beneficial effects on SCI patients, as it can reduce post-paralysis muscle atrophy by increasing muscle tone and improve stability during sitting and standing. Moreover, mild spasticity may contribute to the prevention of post-paralysis osteoporosis and deep vein thrombosis [10]. However, as the severity of spasticity increases, adverse effects such as pain and sleep disturbances may arise [11]. Furthermore, severe spasticity can lead to abnormal trunk posture, significantly limiting patients’ activities of daily living (ADL) and impacting their quality of life (QOL) [2,12].

In conclusion, the detrimental effects of post-SCI spasticity far outweigh its potential benefits. Consequently, it is essential to implement early intervention and effectively control the progression of spasticity at its onset. The elucidation of physiological and pathological alterations in motor neuronal circuitry following SCI constitutes a fundamental prerequisite for understanding the neurobiological foundations of spasticity pathogenesis. A systematic investigation into the temporal dynamics of motor neuron hyperexcitability, particularly the maladaptive plasticity involving altered neurotransmission and synaptic reorganization within spinal reflex arcs, provides critical insights into the progression from initial hyperreflexia to chronic spasticity. Furthermore, delineating the molecular cascades underlying persistent inward currents and voltage-gated ion channel dysregulation enables the identification of novel therapeutic targets for modulating pathological muscle tone.

In previous literature reviews, motor neuron alterations and changes in electrical activity have typically been examined as distinct research domains [13,14]. The present review introduces a significant innovation by systematically integrating the relationship between motor neuron changes and muscle manifestations following SCI. A comprehensive analysis is provided of the multi-level alterations along the brain–spinal cord–muscle motor neuron pathway, as well as the cascading effects of neuronal electrical activity, excitability, and muscle activity. Through the construction of this holistic theoretical framework, the integrative mechanisms of motor neuron systems in the pathogenesis of spasticity post-SCI are elucidated. Furthermore, the mechanisms by which these integrations ultimately lead to abnormal muscle contraction activities are revealed. This integrative analysis is anticipated to establish a more comprehensive theoretical foundation for future research on spasticity treatment, while simultaneously offering novel insights for the optimization of clinical intervention strategies.

## 2. Hyperexcitability of Motor Neurons: The Core Mechanism Underlying Post-SCI Spasticity

### 2.1. H-Reflex and Spasticity Manifestations

Motor neurons function as the final common pathway in skeletal muscle control, coordinating the regulation of muscle contraction and relaxation. The hyperexcitability of motor neurons within the spinal H-reflex circuit contributes to the development of spasticity [15]. This aberrant neuronal activity is modulated through multiple interacting mechanisms. Specifically, PICs have been identified as critical amplifiers of synaptic inputs, while membrane depolarization has been shown to lower action potential thresholds. Notably, alterations in synaptic membrane properties are characterized by three key phenomena: (1) dysregulated expression and activity of neurotransmitter-associated receptors [16], (2) functional remodeling of voltage-gated ion channels [17], and (3) impaired development of dendritic spines on motor neurons. Furthermore, a progressive decline in inhibitory synaptic inputs has been documented to exacerbate neuronal [18] hyperexcitability [19]. Collectively, these interdependent mechanisms establish a self-reinforcing cycle of pathophysiological signaling that sustains spastic motor outputs.

The H-reflex serves as a neurophysiological indicator for evaluating synaptic inhibitory states [20]. In populations exhibiting spasticity, the elevated ratio of potentials produced through electrical stimulation of sensory and motor axons (H_max_/M_max_) has been pathognomonically observed, reflecting amplified excitability within the motor neuron population. Mechanistically, the reflex-dependent depression (RDD) phenomenon of the H-reflex provides quantifiable evidence for assessing both spinal reflex pathway integrity and inhibitory regulation efficacy [21].

The H-reflex has been widely recognized as a principal neurophysiological tool for investigating the causal relationship between motor neuronal hyperexcitability and spasticity manifestations. It has been validated as the gold-standard quantitative method for assessing motor neuron activity [22,23]. Emerging evidence reveals that RDD parameter analysis constitutes a novel biomarker, providing pathophysiological insights into spinal disinhibition mechanisms underlying neuropathic pain development [24]. The decline in H-reflex amplitude is attributed to the enhanced transmission at the Ia fiber–motor neuron junction below the SCI [25]. Concomitantly, diminished RDD magnitudes have been etiologically correlated with supra-threshold motor neuron excitation levels, thereby consolidating the theoretical framework linking neuronal hyperexcitability to spasticity pathogenesis.

In advanced-stage SCI patients, the amplitudes of the M-wave and flexor reflex are reduced, while the magnitude and persistence of the F-wave (the potential generated by recurrent activation of motor neurons) are significantly increased. Due to the loss of inhibitory signal control in spinal reflexes following injury, neuronal activity manifests as excessive excitation, affecting muscle coordination and limb motor capabilities, and even altering muscle properties. Post-traumatic muscular atrophy, as a prevalent pathological progression, may induce biomechanical reconfiguration of joint angle–torque relationships [26]. Crucially, fiber-type compositional alterations occur in affected musculature. Spastic muscles display less pronounced fiber-type transitions compared to non-spastic counterparts. However, to some extent, animals exhibiting spasticity demonstrate less pronounced changes in muscle fiber types, which allows for the preservation of some motor neurons that normally compose muscle fibers. Such changes in muscle fibers influence the magnitude and duration of PICs on motor neurons [27].

### 2.2. Voltage-Gated Conductances in MN Hyperexcitability Pathogenesis

PICs represent voltage-gated intrinsic depolarizing conductances in motor neurons that serve dual physiological functions: sustaining tonic excitatory drive to neuronal networks and amplifying synaptic inputs through several orders of magnitude. These properties enable motor neurons to generate repetitive firing patterns following brief synaptic activation. PICs are primarily mediated by L-type calcium channels and voltage-gated sodium channels, which generate CaPICs and NaPICs, respectively [28]. Research has demonstrated that low-threshold persistent calcium currents (Cav1.3) and sodium currents are responsible for the spontaneous development of motor neuron activity following chronic SCI. This mechanism induces large, rapidly activated plateau potentials, ultimately leading to the manifestation of spasticity [29]. The long-lasting reflex (LLR) following SCI refers to prolonged motor neuron firing lasting several seconds induced by a single low-threshold stimulus, which is mediated by PICs [30]. PICs exhibit voltage dependence, and their activation can be inhibited by hyperpolarization. However, under physiological conditions, dorsal root stimulation only generates transient excitatory postsynaptic potentials (EPSPs < 1 s) [31]. The intact CNS maintains PIC regulation through brainstem monoaminergic modulation [32,33]. After SCI, the diminished PICs lead to a compensatory augmentation of monoaminergic-mediated PIC activation. Following SCI, the loss of descending inhibition induces prolonged EPSP duration after transient sensory stimulation. This results in uncontrolled high motor neuron firing rates in the spinal cord, which are associated with self-sustained firing caused by persistent abnormal activation of PICs. These mechanisms likely contribute to the persistent hyperexcitability of motor neurons [34]. Alterations in the intrinsic properties of spinal motor neurons compromise presynaptic inhibition and reciprocal inhibition, thereby inducing spontaneous motor neurons discharge that ultimately lowers reflex thresholds [35].

Under physiological conditions, inhibitory and excitatory neurotransmitters undergo orderly binding with their respective receptors, precisely regulating both monosynaptic and polysynaptic reflexes at the spinal level. This mechanism delicately maintains the equilibrium between neuronal excitation and inhibition. However, spinal injury profoundly disrupts this homeostasis, leading to an imbalance between excitatory and inhibitory inputs. Consequently, such dysregulation results in dyscoordinated muscle contractions [36]. Synaptic inhibition is categorized into presynaptic and postsynaptic subtypes. Presynaptic inhibition attenuates neurotransmitter release from Ia presynaptic terminals, thereby suppressing α-motor neuron excitability. Conversely, postsynaptic inhibition encompasses Ia inhibition, Ib inhibition, and recurrent inhibition, which collectively modulate stretch reflex excitability. Following SCI, both presynaptic and postsynaptic inhibition pathways exhibit functional impairment. These deficits synergistically contribute to motor neuron hyperexcitability and elevated muscle tone [37].

**Figure 1:**
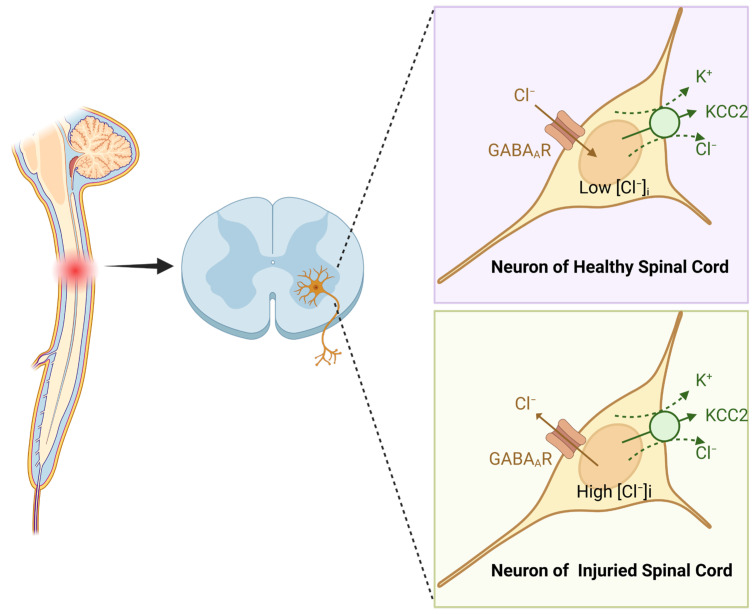
**Structural and ionic changes in spinal neurons under physiological and injury conditions. Left:** schematic representation of spinal cord architecture, highlighting transverse sections and the spatial organization of neuronal populations. **Right (upper and lower panels):** illustration of ionic homeostasis in spinal neurons under normal and post-injury conditions. (Created in https://BioRender.com).

The regulatory roles of GABAA receptors (GABAAR) and K^+^-Cl^−^ cotransporter 2 (KCC2) in motor neurons critically govern postsynaptic inhibition through chloride ion homeostasis modulation within neuroregulatory circuits, fundamentally determining motor neurons excitability profiles. Functioning as a chloride-extruding transmembrane protein, KCC2 mediates Cl^−^ efflux while coordinating GABAergic/glycinergic inhibitory signaling, thereby establishing mechanistical integration with cellular inhibition pathways [38]. Following SCI, both functional efficacy and expression levels of these proteins are markedly reduced, creating enhanced facilitatory conditions for MN excitation [39]. Notably, GABAAR and KCC2 on motor neurons within the lumbar enlargement exhibit a predominant association with post-SCI spasticity pathogenesis. KCC2 downregulation further compromises GABAAR expression, diminishing postsynaptic inhibition efficacy and reducing IPSP generation. This cascade amplifies MN action potential firing frequency and duration [40] (see Figure 1). 

During the subacute phase of SCI, motor neurons innervating both extensor and flexor muscle groups exhibit heightened excitability. This electrophysiological amplification is mechanistically associated with the aberrant activation of PICs, attenuation of synaptic inhibition, and upregulated expression of glutamatergic receptors, collectively contributing to the pathogenesis of muscle spasticity. Numerous therapeutic approaches have been developed by modulating the excitability of motor neurons to alleviate spasticity after SCI, thereby offering patients effective rehabilitation pathways (see Table 1).

The upregulated expression of α-amino-3-hydroxy-5-methyl-4-isoxazolepropionic acid receptor (AMPAR) and N-methyl-D-aspartate receptor (NMDAR) on motor neurons post-injury may contribute to the pathogenesis of early post-traumatic muscle spasticity. NMDAR triggers plateau potentials and self-sustained discharges in motor neurons, ultimately leading to the activation of involuntary muscle spasticity [41].

**Table 1:** Summary of studies on changes in motor neurons following pharmacological inhibition of spasticity post-SCI.

Ref.	Experimental Animals	Experimental Models	Treatment		The Changes in Motor Neurons Following Pharmacological Inhibition of Spasticity Post-SCI	
[42]	Adult female Sprague-Dawley rats	Spinal Contusion Injury at T8/T9	Intraspinal microstimulation		Following SCI, motor neurons exhibit hyperexcitability, resulting in spasticity.	The spatial distribution of spinal responses to sensory feedback increases dramatically in the animals with chronic SCI, consistent with an overall state of dorsal horn hyperexcitability due to a lack of descending neuromodulatory drive.
[43]	Eight-to-ten-week-old male and female C57/Bl6 mice expressing YFP under a Thy1 promoter	Spinal Contusion Injury at L1–L2	Romidepsin	2.5 mg/kg, i.p., once daily over three days successively on post-SCI days 26, 27, and 28.		Romidepsin treatment restores RDD and attenuates hyperreflexia-associated spasticity following SCI. Romidepsin corrects abnormal dendritic spine density on motor neurons associated with SCI and hyperreflexia. Romidepsin primarily normalizes motor neuron thin spine length and head width after SCI.
[44]	Adult C57BL/6J mice of both sexes	Spinal Contusion Injury at T10; Spinal transection injury at T10	STR, PTX	1 µM and 10 µM, respectively.		The development of spasm-like activity following SCI can be caused by changes in synaptic excitation–inhibition balance and/or motoneuron excitability. Upregulated excitatory synaptic inputs and increased motoneuron excitability may be the reason that the evoked spasm activity showed an adaptation in duration.
[45]	Eight-to-ten-week-old male and female mice (c57/bl6)	Spinal Contusion Injury at L1–L2				GLT-1–PSD-95 tripartite synapses in lamina IX ventral horn motor pools show a significant increase in the ventral spinal cord motor regions of animals exhibiting SCI-induced hyperreflexia.
[46]	Selective Rac1KO in astrocytes using a cre-lox system (GFAP-cre/Rac1flox/flox) mouse	Spinal Contusion Injury at L1–L2				Astrocytic Rac1KO reduces injury-induced evoked H-reflex excitability, partly decreases dendritic spine dysgenesis on α-motor neurons, and elevates expression of homeostatic glutamate clearance mechanism, GLT-1.
[47]	Adult C57BL6/J female mice	Complete spinal cord transection at T10	Strychnine, Bicuculline, α-5-HT, Citalopram, Nimodipine	Strychnine at 5 μM for glycine receptors. Bicuculline at 10 μM for GABAA receptors. α-5-HT at 5 μM. Citalopram at 0.3 μM. Nimodipine at 50 and 100 μM.	Crucial ion channel receptors modulate the excitability of motor neurons	Spasm development in SCI is due to enhanced excitability in the motor system through multiple cellular mechanisms. Cav1.3 channels play an important role in the enhanced excitability, and there is a possibility that CPT can be a novel therapy for SCI-induced spasms.
[48]	Adult female Sprague-Dawley rats	Spinal Contusion Injury at T9–T10	NMD	10 mg/kg subcutaneous injection. Starting 1 h after injury for 6 weeks.		Rats treats with NMD showed improvements in locomotion, pain-related behaviors, and spasticity-like symptoms. NMD-treated rats show improvements in KCC2 expression in lumbar motor neurons.
[49]	1.5–2 months old and 180–200 g male Wistar rats	7-Day Rat Hindlimb Suspension (The origin of this activity is somewhat akin to muscle spasticity after spinal cord injuries and is the result of KCC2 content decline in the spinal cord’s motor neurons)	CLP-290	100 mg/kg, i.p.		After 7 days of hindlimb suspension, atrophy of both slow-type and fast-type fibers is observed. The blocking of delayed-onset soleus muscle activity via KCC2 activation leads to the downregulation of AMPK downstream and the upregulation of mTOR targets, but this neither prevents nor enhances atrophy of the soleus muscle or myofiber CSA.
[50]	Adult Wistar Han female rats	Spinal Cord transected at T8/T9	AAV6-GFP-shRNA-CAPN1and AAV6-GFP-shRNA-scramble	At titrations of 1.05 × 1013 genome copies (GC)/ml and 1.5 × 1013 GC/mL, respectively, i.t.		Downregulation of calpain1 in lumbar motoneurons post-SCI effectively prevents the pathological decrease in KCC2 in sublesional transduced motoneurons. Knockdown of calpain1 in lumbar motoneurons reduces spasticity after spinal cord injury in adult rats.
[51]	Adult female Sprague Dawley rats	Spinal transection injury at T12	Bumetanide	30 mg/kg, started 1d post-SCI until the day preceding the terminal experiment (~6 weeks).		After SCI, chloride homeostasis is dysregulated over time in parallel with the decrease in presynaptic inhibition of Ia afferents and postsynaptic inhibition of motoneurons and the development of spasticity. A prolonged bumetanide treatment increases postsynaptic inhibition by hyperpolarizing E_IPSP_ of motoneurons from chronic SCI rats.

## 3. Differential Remodeling of Motor Neurons in Hyperreflexia Development

### 3.1. Upper Motor Neurons and Lower Motor Neurons

**Figure 2:**
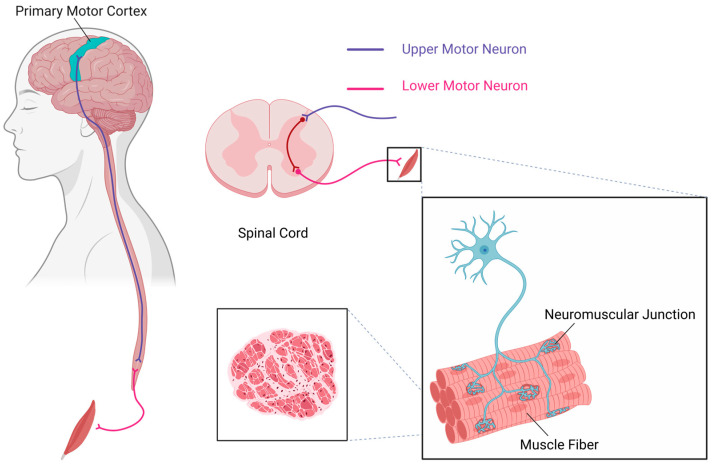
Schematic of UMNs and LMNs pathways. This figure illustrates the anatomical and functional relationship between UMNs and LMNs in the motor pathway. UMNs arise from the primary motor cortex and descend through the spinal cord, establishing synaptic connections with LMNs located in the ventral horn. LMNs subsequently project directly to muscle fibers via the neuromuscular junction (NMJ), transmitting signals to trigger muscle contraction. (Created in https://BioRender.com).

Motor neurons reside within the central nervous system (CNS) and are responsible for generating movements essential for life. These neurons regulate extrinsic skeletal muscle activity by driving contractions, thereby mediating the initiation, modulation, and regulation of voluntary movements. As one of the most vital neuronal subtypes in vertebrates, distinct motor neuron populations exist in defined stoichiometric ratios to collectively innervate individual muscles, with their cellular compositions being precisely matched to the functional properties of their target muscles. This organized ensemble of motor neurons constitutes a motor neuron pool [52]. The recruitment gain of motor neuron pools helps modulate motor outputs at the final stage of spinal cord processing and is involved in the reflex excitability changes associated with movement or nervous system injuries [53]. Neural pathways within the CNS ultimately converge onto motor neuron pools, each of which precisely controls a specific muscle group. The synergistic interactions within the CNS play a crucial role in both voluntary movements and reflex behaviors. Experimental evidence confirms the quadriceps femoris motor neuron pool localizes to spinal segments L2–L3, while the gastrocnemius motor neuron pool resides in L4–L5; both pools exhibit coordinated activation during the stance phase of bipedal locomotion [54,55,56] (see Figure 2). 

#### 3.1.1. The Role of Upper Motor Neurons in Motor Control and Dysfunction Following Injury

Upper motor neurons (UMNs), which form the crucial connection between the cerebral cortex and the spinal cord, play a pivotal role in transmitting cortical inputs to the spinal cord. These neurons are indispensable for the initiation and modulation of voluntary movement [57]. Following UMN lesions, patients commonly exhibit motor dysfunction, decreased reflex stimulus sensitivity, and spasticity [58]. Generally, spasticity and spastic states are recognized as common outcomes following UMN injury, likely due to abnormal sensory input in the spinal cord after SCI, which induces exaggerated stretch reflexes. This process leads to hyper-reflexive activation of α motor neurons. Spasticity, hyperreflexia, and clonus following UMN lesions share a common characteristic of muscle hyperactivity. The motor cortex output signals inhibit motor neurons innervating antagonistic muscles through Ia interneurons.

Degeneration of UMNs has been associated with the occurrence of hereditary spastic paraplegia (HSP), primary lateral sclerosis (PLS), and amyotrophic lateral sclerosis (ALS) [59]. Restoring plasticity in corticospinal motor neurons may enhance voluntary control of targeted muscles in patients with SCI [60]. Despite the occurrence of SCI, a significant proportion of corticospinal motor neurons are preserved during the acute phase. As a result, the cerebral modulation of spinal reflexes is maintained. Axonal pathways from upper UMNs are capable of transmitting signals across the lesion site, thereby influencing motor activities distal to the injury. Furthermore, repetitive external stimulation of spinal reflex circuits has been proposed as a potential therapeutic strategy to activate residual functions of lower motor neurons (LMNs) following injury [61].

#### 3.1.2. Multiple Inputs to Lower Motor Neurons and Their Role in Motor Control

LMNs receive inputs from UMNs, sensory neurons, and interneurons. They are modulated by the corticospinal tract and are also regulated by various synaptic pathways, including those from the vestibular nuclei, tectal nuclei, and red nucleus [62]. LMNs located at distinct positions within the spinal cord precisely regulate the distal or proximal regions of specific muscles [63]. The loss of functional LMNs results in a reduction of electrical activity during maximal muscle contraction, primarily manifesting as muscle atrophy, muscle weakness, and hyporeflexia, without sensory abnormalities [64]. There are numerous disorders that commonly lead to LMN pathology, including spinal muscular atrophy (SMA), spinobulbar muscular atrophy, distal hereditary motor neuropathies (dHMNs), progressive muscular atrophy, poliomyelitis, immune-mediated neuropathies such as Guillain–Barré syndrome (GBS), multifocal motor neuropathy (MMN), and chronic inflammatory demyelinating polyneuropathy (CIDP) [65].

##### Spinal Motor Neurons and Their Pathophysiological Role in Spinal Cord Injury

The activity of motor neurons is not linear. They exhibit a “bistable” characteristic, which is the plateau potential. In spinal motor neurons, this current is primarily mediated by the influx of Ca^2+^ through L-type Ca^2+^ channels of the Ca_(v)_1.3 subtype [66]. The amplitude of the PIC is proportional to the level of neuromodulatory input from the brainstem, which is primarily mediated by monoamines such as serotonin and norepinephrine. In the study by Heckman et al., it was found that PICs are particularly strong in spinal motor neurons. PICs have the capacity to induce neuronal firing with minimal synaptic stimulation. Under physiological conditions, the inhibitory influence exerted by the brainstem can effectively inactivate PICs. However, after SCI, this inhibitory influence is lost, leading to an amplification of PIC’s effects [67]. The abnormal recovery of PICs results in the broadening of movement-related receptive fields (MRRFs). The precise execution of movement is dependent on accurate input to spinal motor neurons, which are activated through the rotation of limb joints. Post-SCI alterations in MRRFs are known to induce joint rotation, thereby triggering the activation of entire limb muscles. This phenomenon may also constitute a potential mechanism contributing to the development of limb spasticity [53].

##### Mechanisms of Elevated Fatigability and Enhanced Twitch Force in Spastic Muscles Post-SCI

Fatigue-sensitive muscle afferent nerves are unable to influence the overall excitability of muscle motor neurons during maximal contraction [68]. During fatigue, input from group III and IV muscle afferent nerves of the ipsilateral or antagonistic muscles inhibits extensor motor neurons while facilitating flexor motor neurons. The significance of muscle fatigue lies in its limitation of muscle drive, preventing unrestrained muscle contraction [69]. In patients with SCI, the contraction properties and force–frequency relationship (FFR) of the paralyzed quadriceps muscles are altered. The paralyzed muscles exhibit faster contraction and relaxation speeds under both non-fatigued and fatigued conditions [70,71].

Muscle strength gradually decreases during physical exertion, a phenomenon termed muscle fatigue [72]. Under physiological conditions, this fatigue is mitigated through the intrinsic adaptive mechanisms of motor neurons [73]. Fatigue-sensitive muscle afferent nerves demonstrate no detectable influence on the global excitability of muscle motor neurons during maximal voluntary contraction [68]. Importantly, group III and IV muscle afferent inputs originating from ipsilateral or antagonistic muscles exhibit differential regulatory effects during fatiguing contractions: extensor motor neurons are inhibited while flexor motor neurons are facilitated. This limitation of muscle drive serves as a critical protective mechanism against sustained excessive muscle contraction [69]. SCI patients exhibit characteristic alterations in both contraction dynamics and the FFR of paralyzed quadriceps muscles. Specifically, faster contraction and relaxation speeds are recorded in these muscles under both pre-fatigue and post-fatigue conditions [70,71].

Harris et al. demonstrated that spastic muscles following SCI exhibit significantly elevated fatigability and enhanced twitch force compared to normal muscles [69]. Muscles below the SCI level undergo profound structural and functional alterations, including severe muscle atrophy, fiber type transition, and impaired blood perfusion, all of which are mechanistically associated with post-injury fatigability. Specifically, SCI induces atrophy of slow-twitch type I fibers while promoting a phenotypic transition toward fatigue-prone fast-twitch type II fibers [74,75]. Additionally, paralyzed muscles [76] demonstrate markedly increased passive tension in fast-type IIx fibers [77]. The persistence of slow-type contractile properties impedes classical slow-to-fast fiber-type conversion, resulting in repetitive or sustained muscle contractions.

### 3.2. α, γ, and β Motor Neurons

Motor neurons are classified into three distinct types: α-motor neurons (αMNs), β-motor neurons (βMNs), and γ-motor neurons (γMNs) [78]. Each type is known to unique roles in the regulation of muscle contraction and differentially contributes to the development of spasticity following SCI. Particular attention has been directed toward αMNs, not only because they are the most extensively investigated in motor function-related research, but also due to the presence of their subtypes, whose specific mechanistic roles in post-SCI spasticity are yet to be fully elucidated. Furthermore, a comprehensive review of the existing literature suggests that the phenotypic transition of αMNs subtypes after SCI may serve as a key pathogenic mechanism underlying spasticity. Therapeutic approaches targeting the inhibition of this transition during the acute phase of SCI, or the modulation of subtype proportions through established therapeutic interventions in the chronic phase, could potentially offer a novel and promising strategy for the management of post-SCI spasticity.

#### 3.2.1. Functional Changes in α-Motor Neurons Post-SCI and Their Association with Muscle Spasticity

αMNs constitute the most predominant class of motor neurons. Functionally, they play a critical role in the regulation of muscle contraction by receiving synaptic inputs via their connections with the neuromuscular junction and skeletal muscles. Specifically, these neurons are primarily responsible for the innervation of extrafusal muscle fibers, which are essential for voluntary motor control [79]. αMNs are categorized into three distinct subtypes: fast-twitch fatigable (αFF), fast-twitch fatigue-resistant (αFR), and slow-twitch fatigue-resistant (αS) [78,80,81]. These subtypes are crucial for the establishment of motor units, which serve as the fundamental units of coordinated muscle activity. Different subtypes of αMNs innervate motor units with distinct physiological properties, resulting in varied contractile characteristics in the muscle fibers they control.

The αS subtype is associated with type I muscle fibers, which are characterized as slow-twitch and fatigue-resistant [82]. The αRF subtype is linked to type IIa muscle fibers, which exhibit fast-twitch and fatigue-resistant properties. In contrast, the αFF subtype corresponds to type IIb muscle fibers, which are fast-twitch but fatigue-prone [74]. αS motor neurons exhibit higher input resistance, which enables them to reach the firing threshold more readily during action potential generation. Additionally, the PIC observed in their dendrites is more sustained compared to that of αFF motor neurons [78]. Furthermore, αS motor neurons are more easily activated and, upon activation, exhibit the longest duration of activity, thereby playing a critical role in sustaining muscle contraction [78].

The soleus muscle is predominantly composed of slow-twitch muscle fibers, which enable it to generate greater contractile force in response to brief stimuli [83]. In contrast, the tibialis anterior muscle is primarily composed of fast-twitch muscle fibers [84,85]. The following section will elucidate the roles and characteristics of the soleus muscle and tibialis anterior muscle following SCI. Subsequently, it will discuss how a specific type of muscle fiber may contribute predominantly to the development of muscle spasticity post-SCI.

Afterhyperpolarization (AHP) is an intrinsic characteristic of motor neurons, mediated by calcium-dependent potassium currents, and plays a deterministic role in regulating their firing frequency [86]. A longer AHP duration indicates that motor neurons can maintain lower-frequency repetitive firing for an [87] extended period. Upon activation, the three distinct subtypes of motor neurons exhibit different AHP durations, which contribute to the variations in contractile properties of the muscles they innervate. Compared to αFF MNs, αS MNs exhibit a longer AHP. The persistent presence of the PIC enables these neurons to sustain activity even in the absence of excitatory synaptic input [50], allowing them to maintain prolonged repetitive firing following a brief depolarizing drive.

The properties of motor units in different regions undergo transformation toward distinct phenotypic characteristics after injury. SCI often renders motor neurons more susceptible to weakness and fatigue, thereby impairing the motor function of the limbs. In cases of spasticity post-SCI, it has been observed that the firing rate modulation of these units exceeds that during voluntary contraction [88,89]. Moreover, following SCI, the balance between excitatory and inhibitory signals in descending inputs is disrupted, leading to a reduction in inhibitory effects, which makes it challenging to terminate motor unit activity. Concurrently, due to the decrease in units under voluntary control post-injury, the recruitment force required for activation increases. The strong PIC is responsible for the increased unit recruitment. This alteration also results in a reduction in the precision of muscle force control. Researchers hypothesize that enhancing the depolarizing current in the motor neuron pool could potentially facilitate voluntary contraction.

Current research on the treatment and mechanisms of spasticity following SCI predominantly focuses on αMNs. The exploration targeting αS MNs and αFF MNs, with the aim of enhancing the facilitation of voluntary contraction in motor units, may hold significant potential for future precise modulation of motor neuron excitability to reduce involuntary muscle contractions and spasticity.

#### 3.2.2. Functional Changes in γ-Motor Neurons Post-SCI and Their Association with Muscle Spasticity

The function of γMNs is to regulate the sensitivity of muscle spindles to muscle stretch [90]. These neurons are critical for motor task execution and maintenance of the equilibrium between muscle contraction and relaxation. They primarily govern the intrafusal muscle fibers, constituting the IF fibers [91], and do not receive signal input from Ia fibers [78]. Muscle spindles provide signals that encode muscle length and the rate of muscle stretch, thereby regulating muscle activity. Muscle spindles encode both static muscle length and dynamic stretch velocity, thereby modulating neuromuscular activity. In contrast to αMNs, γMNs exhibit distinct electrophysiological properties, including smaller somatic dimensions and enhanced intrinsic excitability, while operating independently of afferent synaptic drive [92,93]. While αMNs constitute the principal effectors of skeletal muscle contraction, γMNs play an indispensable role in executing movement, maintaining balance, and sustaining posture, all of which are essential for coordinated muscle activity [94]. During extrafusal fiber contraction, co-activation of γMNs induces IF fiber shortening. This homeostatic mechanism suppresses persistent motor neuron discharge and normalizes baseline muscle tone [95]. This phenomenon may be attributed to the direct or indirect inhibitory or excitatory signal inputs to γMNs, which regulate muscle tension [96]. αMNs and γMNs must maintain a delicate coordination to ensure the rapid and accurate execution of movement and posture.

Therefore, the precise coordination between αMNs and γMNs is a prerequisite for neuromechanical efficiency during movement execution. Consequently, post-SCI therapeutic strategies targeting γMN excitability modulation, α-γ motor neuron synergy restoration, and fusimotor system recalibration may mitigate spasticity arising from discoordinated muscular contractions. The mechanisms underlying the modulation of γMNs and their associated fusimotor system in regulating muscle spasticity and coordinated motor activity following SCI remain poorly understood and insufficiently investigated.

#### 3.2.3. Functional Changes in β-Motor Neurons Post-SCI and Their Association with Muscle Spasticity

**Figure 3:**
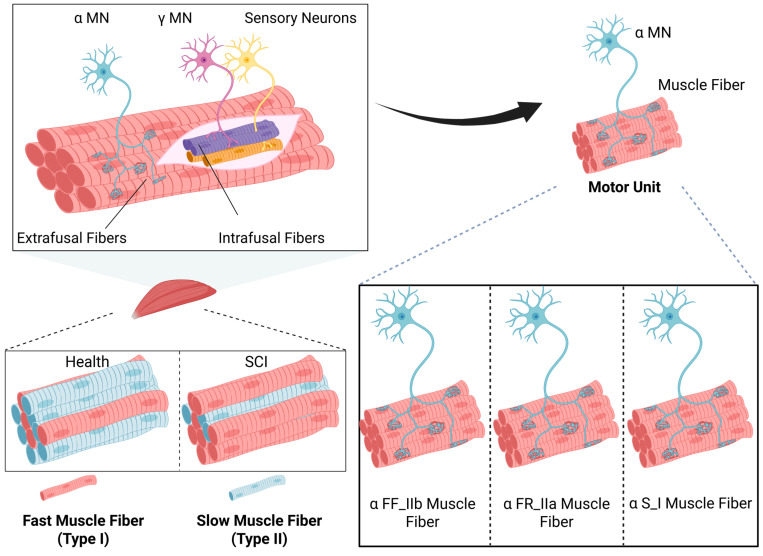
The structure, innervation, and fiber classification of muscle units. **Upper left:** schematic representation of a muscle unit comprising α-motor neurons (α-MNs), γ-motor neurons (γ-MNs), and sensory neurons. α-MNs innervate extrafusal muscle fibers, which are responsible for generating force during muscle contraction. γ-MNs innervate intrafusal fibers located within muscle spindles, modulating their sensitivity to stretch. Sensory neurons wrap around intrafusal fibers and transmit afferent signals that reflect muscle stretch and contraction states. **Lower left:** comparison of muscle fiber morphology and function under healthy and spinal cord injury (SCI) conditions. In healthy muscle, fibers are structurally intact and well organized, with Type I (slow-twitch) and Type II (fast-twitch) fibers working synergistically to support normal muscle function. Following SCI, muscle fibers exhibit atrophy, disarray, and altered proportions of fiber types, contributing to impaired contractile performance. Type II fibers are characterized by larger diameters and rapid, forceful contractions, while Type I fibers possess smaller diameters, slower contraction speeds, and enhanced fatigue resistance. **Upper right:** enlarged view of the muscle unit architecture, emphasizing the spatial distribution of extrafusal and intrafusal fibers. Extrafusal fibers are densely packed and structurally robust, mediating voluntary muscle contractions. Intrafusal fibers, embedded within muscle spindles, detect changes in muscle length and tension via terminal sensory nerve endings. **Lower right:** structural and innervation characteristics of three major muscle fiber subtypes: fast-fatigable (FF, Type IIb), fast fatigue-resistant (FR, Type IIa), and slow (S, Type I) fibers. FF fibers have large diameters and contract rapidly but fatigue quickly; FR fibers display intermediate size and contraction speed with moderate resistance to fatigue; S fibers are small, contract slowly, and sustain prolonged activity. (Created in https://BioRender.com).

Research on βMNs remains limited, and their functional role has not yet been fully elucidated. Emerging evidence suggests that βMNs may exhibit dual innervation of both intrafusal IF fibers and extrafusal EF fibers [97]. Both muscle activity and sensory feedback from muscle spindles are regulated by βMNs. Additionally, during extrafusal muscle fiber contraction, βMNs are co-activated, potentially contributing to the extreme contraction of intrafusal fibers, which facilitates tension restoration. Additionally, during extrafusal muscle fiber contraction, βMNs are co-activated, potentially contributing to the extreme contraction of intrafusal fibers, which facilitates tension restoration. Such functionality of βMNs highlights their potential significance in motor coordination and postural stability. However, the neurophysiological mechanisms underlying these processes remain poorly understood, necessitating further investigation into their synaptic connectivity, electrophysiological properties, and functional contributions to the fusimotor system (see Figure 3). 

## 4. Discussion

In this article, we reviewed the literature concerning motor neuron alterations after SCI and therapeutic strategies targeting motor neurons. By elucidating the pathophysiological changes in motor neurons post-SCI, we underscored their pivotal role in the development and management of post-SCI spasticity. As the final integrators of motor commands, MNs undergo profound dysregulation following SCI, characterized by three hallmark changes: (1) hyperexcitability due to altered voltage-gated ion channel expression, (2) sustained plateau potentials from persistent inward currents, and (3) apoptotic cascades triggered by neurotrophic factor deprivation. These changes contribute significantly to the hypertonia and spasticity observed in patients.

Despite preclinical promise, clinical translation remains constrained by two fundamental challenges: the network complexity of MN subtypes and lesion heterogeneity across SCI patients. Our review may reveal that current strategies predominantly target αMNs at the injury epicenter, neglecting the critical contributions of βMNs and γ-MNs. The latter’s role in maintaining γ-loop homeostasis through dynamic control of intrafusal fiber tension is particularly understudied, despite its direct impact on spindle afferent signaling and long-latency stretch reflexes.

Equally overlooked are supraspinal MNs in motor cortex/brainstem, whose corticospinal tract degeneration disrupts top-down modulation of spinal circuits. Muscle motor neurons, located downstream of spinal motor neurons, are significantly affected by the loss of trophic support post-SCI. Their dysfunction contributes to muscle atrophy, spindle degeneration, and impaired contractile properties, which exacerbate spasticity. Addressing these changes may offer a novel therapeutic avenue for alleviating post-SCI spasticity.

The core of post-SCI spasticity treatment lies in preventing motor neuron apoptosis and restoring their normal firing patterns. Functionally, this involves correcting abnormal muscle contractions and improving the regulation of muscle activity [95]. However, it is crucial to recognize that SCI patients often experience muscle atrophy and structural changes in muscle spindles, such as alterations in spindle number and length. These changes further impair muscle relaxation and contraction, highlighting the need for comprehensive therapeutic strategies that target both neuronal and muscular components.

## 5. Conclusions

In conclusion, mitigating the onset and progression of post-SCI spasticity requires a deeper and more holistic understanding of the underlying mechanisms. Current research remains insufficient to support the development of novel pharmacological and therapeutic interventions. Future studies should focus on elucidating the roles of different motor neuron subtypes, their interactions with muscle spindles, and the impact of supraspinal inputs. Additionally, exploring the potential of neuroprotective agents, activity-based therapies, and neuromodulation techniques may pave the way for more effective treatments.

## Data Availability

Not applicable.

## Abbreviations

The following abbreviations are used in this manuscript:

SCI	spinal cord injury
PICs	persistent inward currents
ADLs	activities of daily living
QOL	quality of life
LLR	long-lasting reflex
EPSP	excitatory postsynaptic potential
KCC2	K+-Cl− cotransporter 2
GABA	γ-aminobutyric acid
IPSP	inhibitory postsynaptic potential
AMPAR	α-amino-3-hydroxy-5-methyl-4-isoxazolepropionic acid receptor
NMDAR	N-methyl-D-aspartate receptor
NMD	nimodipine
PA	proprioceptive afferent
GABApre	GABAergic inhibitory presynaptic regulation of 1A terminals
RDD	rate-dependent depression
STR	Strychnine
PTX	picrotoxin
GLT-1	glutamate transporter-1
PSD-95	postsynaptic density protein 95
SCT	spinal cord transection
GL	Gastrocnemius lateralis
TA	Tibialis anterior
aSCI	acute spinal cord transections
CNS	central nervous system
UMN	upper motor neuron
LMN	lower motor neuron
HSP	hereditary spastic paraplegia
PLS	primary lateral sclerosis
ALS	amyotrophic lateral sclerosis
SMA	spinal muscular atrophy
dHMNs	distal hereditary motor neuropathies
GBS	Guillain–Barré syndrome
MMN	multifocal motor neuropathy
CIDP	chronic inflammatory demyelinating polyneuropathy
MRRF	movement-related receptive field
FFR	force–frequency relationship
αMN	α-motor neuron
βMN	β-motor neuron
γMN	γ-motor neuron
αFF	fast-twitch fatigable
αFR	fast-twitch fatigue-resistant
αS	slow-twitch fatigue-resistant
AHP	afterhyperpolarization
